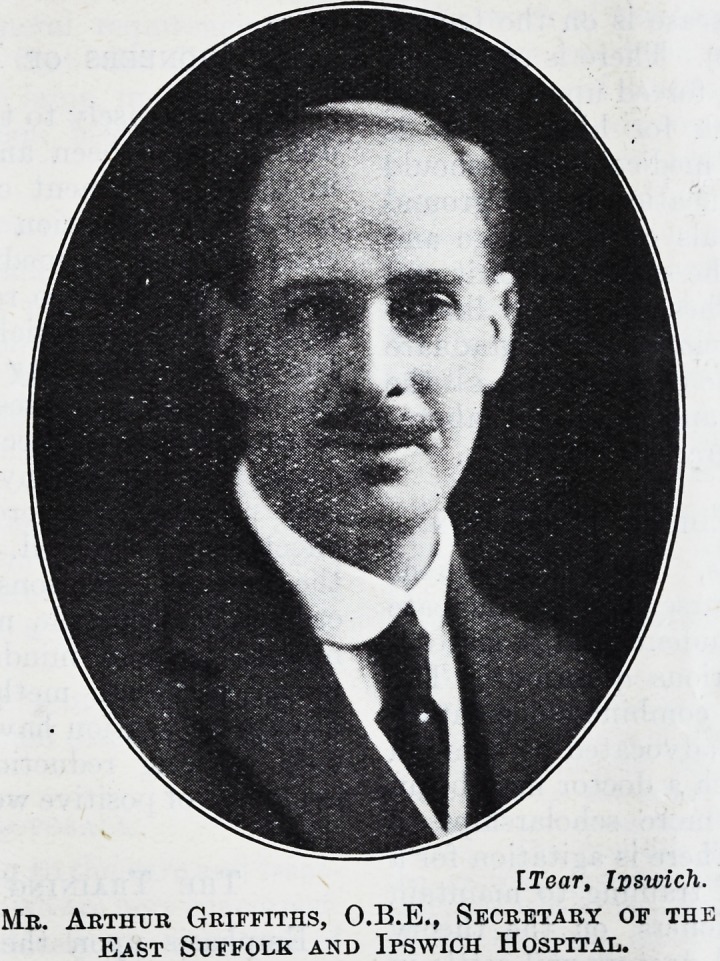# Hospital Men of Mark

**Published:** 1924-03

**Authors:** Arthur Griffiths

**Affiliations:** O.B.E., of the East Suffolk and Ipswich Hospital


### HOSPITAL MEN OF MARK.

### MR. ARTHUR GRIFFITHS, O.B.E., OF THE EAST SUFFOLK AND IPSWICH HOSPITAL.

THE East Suffolk and Ipswich Hospital has entered upon the eighty-eighth year of its existence. For three-quarters of a century after its foundation the Hospital world knew little of it or of its work, during the past few years it has rapidly come to the front and is now numbered among the first half-dozen provincial hospitals without medical schools. Its secretary (Mr. Arthur Griffiths) has been very closely associated with this development. Like several others who have "made good" in the Hospital profession, Mr. Griffiths was an outsider. He is a Fellow of the London Association of Accountants, and when offered the appointment was holding the office of accountant to a local authority. Mr. Griffiths is a strong believer in the principle of purchasing in the wholesale markets, and will not give way to the desire of subscribing retail traders to participate in the hospital's trading. For the past ten years, on a total turnover of £400,000, he has done the buying for his hospital without contracts. But he has an equally strong belief in the value of accurate statistics of expenditure in all departments, and of the necessity of keeping his Committee well informed on matters of hospital economics.

He prides himself on the assistance he is able to render officials of other hospitals, especially the smaller institutions, and every query submitted to him by his fellow-secretaries receives a prompt and full answer. No doubt this accounts in some measure for the fact that he was appointed Honorary Secretary of the Voluntary Hospitals Committee for the County of Suffolk, Honorary Secretary to the Regional Committee of the British Hospitals Association and a member of the Council of that body.

Mr. Griffiths has made it his business to acquaint himself with the plans of construction and methods of management of some of the principal hospitals in this country and—in a recent tour of America—of the modern hospitals in the States and Canada. During the Great War he undertook the administration of his own hospital, enlarged by temporary accommodation for the reception of wounded, and four other subsidiary hospitals all situated in that part of the East Coast which suffered so severely from enemy raids. For these services he was awarded the O.B.E. and la Medaille du Roi Albert.

Mr. Griffiths has varied a life of strenuous work with sports of all kinds, fishing and aquatics having specially appealed to him. His holidays have been spent in walking and climbing tours in Switzerland and cycling tours in this country until a few years since, when he became an ardent motorist. In indoor recreations he has taken an active part in musical societies for many years. One of his *confrères*, on being asked what he knew of Mr. Griffiths, replied that he knew him "as a man who held the most offices in Freemasonry and played the best game of billiards of any hospital secretary in the kingdom." He has always taken a deep interest in building and engineering schemes, was at one time a director of an electric supply company, and is now a director of a gas company. The knowledge he has acquired in this and other commercial concerns has no doubt been of great service to his Board.

[*Tear, Ipswich.*

MR. ARTHUR GRIFFITHS, O.B.E., SECRETARY OF THE EAST SUFFOLK AND IPSWICH HOSPITAL.

At the termination of the Great War, Mr. Griffiths prepared an extensive scheme for the enlargement and modernisation of his hospital. This scheme was approved by the Board and the medical staff and adopted by the Borough and County Committees as the object of their respective War Memorials. The work is now in progress at the estimated cost of £65,000, £62,000 of which has already been subscribed. Mr. Griffiths has made a special study of the construction, equipment and management of Convalescent Homes in conjunction with General Hospitals, and the Hospital's new Convalescent Home now in course of construction at Felixstowe, at a cost of £100,000, including land, promises to be one of the finest buildings of its type in the country. He attributes any success he may have achieved to the hearty support he has received from the Chairmen of his Committees, the members of his Board, and especially from his Chairman. Mr. W. B. Elkington, the Chairman of the East Suffolk and Ipswich Hospital, has served that hospital as it lies in the power of but few Chairmen to serve their institution. He has now entered on his third year of chairmanship, after having previously served nine years as Vice-chairman, and the unique position the hospital holds, both in respect of the work of the central institution and its Convalescent Home Scheme, which is fully endowed, speaks volumes for the work of the Board of Management in general and in particular for the Chairman. As Editor and managing director of the Ipswich newspapers, Mr. Elkington is in a position to give advice and render great assistance to the institution which has adopted him as its leader.

## Figures and Tables

**Figure f1:**